# Tuberculosis treatment outcomes of non-citizen migrants: Israel compared to other high-income countries

**DOI:** 10.1186/s13584-020-00386-1

**Published:** 2020-08-03

**Authors:** D. Chemtob, E. Ogum

**Affiliations:** 1 Department of Tuberculosis and AIDS, Ministry of Health, P.O.B. 1176, 944727 Jerusalem, Israel; 2 Braun School of Public Health and Community Medicine, Hebrew University of Jerusalem, Jerusalem, Israel

**Keywords:** Comparative study, Migrant health, Tuberculosis

## Abstract

**Abstract:**

Background: In TB low incidence countries**,** the outcome of TB treatment among non-citizen migrants from endemic countries affects ability to eliminate TB. This study compares TB treatment outcomes among non-citizen migrants in select pre-elimination country based on their policies for non-citizen migrant TB patients in order to determine how policy affects TB outcomes.

**Methods:**

A literature review was conducted via PUBMED, MEDLINE (2000–2017) on TB policy among non-citizen migrants and treatment outcome. Treatment outcome among migrants diagnosed in Israel during 2000–2014 was analysed.

**Results:**

In total, 18 publications met the inclusion criteria. All the countries reviewed except the United States offered free TB treatment to undocumented migrants. Successful TB treatment outcome for non-citizen migrants in Israel was 87%, the Netherlands was 90.7%, the UK was 82.1%, and outcomes in the US and Australia were not published.

**Conclusions:**

There is a need to standardize results based on international definitions of migrants, asylum seekers, and refugees in order to determine status-specific barriers and to facilitate international comparisons. Policies insuring free access to TB care for non-citizen migrants are an important element for TB elimination in low incidence countries.

## Introduction

Immigration policies and shifting migration patterns over the past five decades have brought larger numbers of permanent and temporary migrants from regions of the world with a high incidence of active TB to areas with a low incidence [1]. Nearly 50% of TB cases in low-incidence countries are among foreign-born residents and citizens [1]. Among the 33 countries defined as being in the pre-elimination phase by the WHO in 2015, the proportion of foreign-born TB cases notified in 2012 for selected countries were as followed: Israel – 90%; Australia – 87%; The Netherlands – 73%; and the USA – 63% [2]. The United Kingdom, while not in the pre-elimination phase, has 73% of tuberculosis incidence among foreign-born migrants. However, policies regarding TB management for migrants in these countries are not clearly stated, especially for migrants without official legal status [1].

Trends in foreign-born TB cases vary across countries. In Israel, studies focused on migrants who obtained Israeli citizenship, as non-citizen migrants were a marginal proportion of the population prior to mass migration from high-endemic countries beginning in 2006 [3–6]. Australia’s proportion of TB in foreign-born people has risen steadily over the past 15 years, despite decreases in overall incidence [2, 7]. Since 2000, migrants to Australia have contributed to more than 80% of TB cases [2, 7]. In the Netherlands, TB incidence is closely linked to the inflow of migrants and asylum-seekers [8, 9]. TB cases reported in the UK were 15 times higher among foreign-born migrants than the UK-born population [10]. In 2016, foreign-born individuals accounted for 66.2% of the reported cases in the US [11]. In the same year, the US saw an increase in the number of TB cases, the second time in 23 years the rate did not decline.

In these countries, migration presents a significant challenge for TB surveillance and control, particularly due to the tentative or undocumented status of many migrants. Subsequently, data specific to undocumented migrants are often missing or underestimated. Policies in low TB incidence countries are often imprecise concerning care for undocumented migrants, and sometimes for refugees, asylum seekers, and otherwise classified migrants [11–13]. In this study, we explore the current policies of five high-income countries regarding TB patients who are non-citizens of these countries.

### Definitions

Definitions related to migrants used in this article are similar to the ones used elsewhere as defined by specialized international organizations [14, 15]. The overall definition of a migrant is ‘any person who is moving or has moved across an international border or within a State away from his/her habitual place of residence, regardless of 1) the person’s legal status; 2) whether the movement is voluntary or involuntary; 3) what the causes for the movement are; or 4) the length of the stay’ [14, 15]. ‘Undocumented migrants’ were defined as migrants who do not have the necessary documentation to enter or remain legally in a country [14, 15]. An ‘asylum seeker’ was defined as a person who seeks safety from persecution or serious harm in a country other than his or her own and awaits a decision on the application for refugee status. A ‘refugee’ was defined as a person who has been forced to flee his or her country because of persecution, war or violence and seeks protection in another country [14, 15].

In order to encompass both documented migrants without citizenship (such as labor migrants, asylum seekers, and refugees) and undocumented migrants, we use the term “non-citizen migrants” in this article.

Pre-elimination for TB has been defined by several international bodies, including the WHO. Until 2035, the WHO global strategy milestone for TB elimination is 10 cases per 100,000 people [2].

However, for pre-elimination countries (< 100 cases per million population), this definition is irrelevant. The current goal for such countries is less than 1 smear-positive case per million population [2].

Treatment success for treatment-sensitive cases was defined as a patient who completed treatment and who either had a smear or culture negative test or who completed treatment but did not undergo a smear or culture test in the last month [3].

## Methods

Five high-income countries, 4 of which had a significant proportion of foreign-born cases as notified in 2012 were selected, and a search of multiple databases (The Cochrane Database of Systematic Reviews, MEDLINE and EMBASE) was conducted in order to identify relevant articles on TB control policy among non-citizen migrants and TB treatment outcome published between January 2000 to August 2017. We used keywords in various combinations: ‘tuberculosis OR TB’ AND ‘Non-citizen’ OR ‘undocumented migrants’ OR ‘migrants’ OR ‘refugees’ OR ‘asylum seekers’ OR ‘foreign-born’ AND ‘policy’. Articles for which at least an English abstract was available were included.

In addition, TB treatment outcome among migrants diagnosed in Israel for the years 2000–2014 were analysed retrospectively. Data were used from the Israeli National Registry of TB at the Department of Tuberculosis and AIDS, Ministry of Health (MoH). Data from the 4 other countries for similar years were extracted from the literature or were sought through contacting medical officers in charge of TB control and/or surveillance in these countries (Dr. Gerard de Vries for the Netherlands & Dr. Maeve Lalor for the UK), and/or through the national website (for Australia and US). Incidence rate and TB outcomes were also determined. The data obtained were analyzed using IBM SPSS version 20.

## Results

**Fig. 1:**
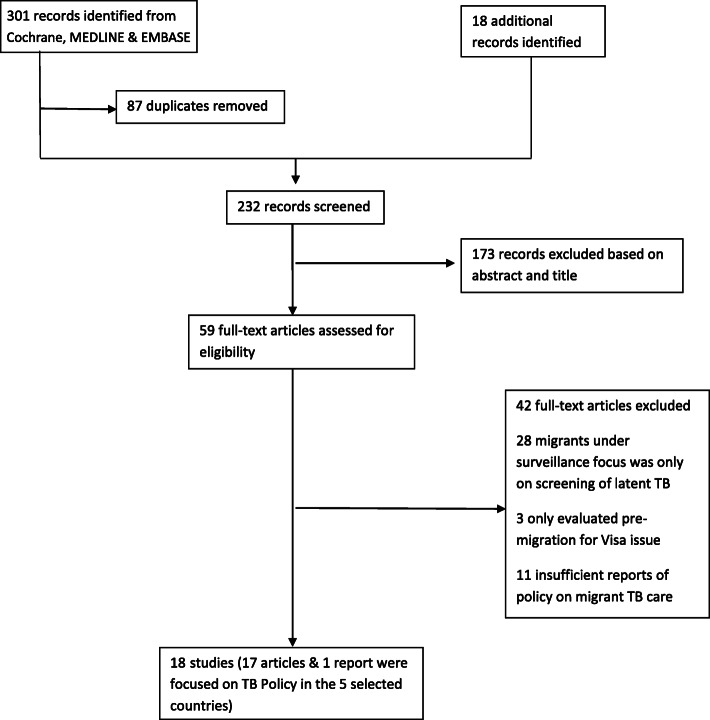
Literature selection search on TB policy for the non-citizens of the 5 selected western countries (Australia, Israel, The Netherlands, UK and USA)

Three hundred and nineteen articles and abstracts written in English, French, Spanish, Italian and Portuguese were considered. After screenings of the titles and abstracts, 59 studies were reviewed and 18 articles were used to generate the results (see Table 1 & Fig. 1). Articles that were not focused on TB Policy were excluded.

**Table 1:** Articles reviewed on TB control policy among migrants in relation to the selected countries

Authors	Year of publication	Study design	Receiving country	Reference
Alvarez GG. et al	2011	Descriptive study of immigration TB screening programs	EU/EEA & Non-EU/EEA	[16]
Chemtob D. et al	2003	Policy and Record review	Israel	[3]
Chemtob D. et al	2003	Retrospective cohort analysis and Interview	Israel	[4]
Chemtob D. et al	2015	Retrospective analysis and records review	Israel	[17]
Correa-Velez I. et al	2005	Policy review	Australia	[18]
Dara M. et al	2012	Systematic review	EU/EEA & Non-EU/EEA	[11]
Dara M. et al	2016	Cross-sectional study	EU/EEA	[12]
Dara M. et al	2017	Non-systematic literature review	EU/EEA & Non-EU/EEA	[19]
Eziefula AC. et al	2014	Retrospective records review	UK	[20]
Hodge JG. et al	2009	Policy review	USA	[21]
Kik SV. et al	2009	A cross-sectional survey	The Netherlands	[22]
Kronfol NM. et al	2013	Retrospective records review	EU/EEA & Non-EU/EEA	[1]
Kunst H. et al	2017	Systematic review	EU	[13]
La’Marcus TW. et al	2015	Retrospective records review and Cost-effectiveness	USA	[23]
Lönnroth K. et al	2015	A narrative review of WHO policy documents and guidelines, as well as published literature	EU/EEA & Non-EU/EEA	[2]
Lönnroth K. et al	2017	A narrative literature review and secondary data analyses	EU/EEA & Non-EU/EEA	[24]
Odone A. et al	2014	Comprehensive literature review	EU/EEA	[25]
van den Bosch C. et al	2000	Retrospective records review	UK	[26]

### Policy recommendations

As of 2014, universal health coverage policies and regulatory frameworks for TB notification were recommended by the WHO end-TB strategy. In this recommendation, it was emphasized that TB diagnosis and treatment must be free of charge for all migrants irrespective of a patient’s legal status in the country [24, 27]

#### National Policy: Israel

Since 1997, the National TB Control Programme has provided diagnosis and treatment to patients with TB without charge regardless of citizenship or migration status [4, 17]. For all TB cases, an epidemiological enquiry is performed for active case finding among close contacts of the diagnosed patient [16, 17].

#### National Policy: Australia

TB treatment is available without charge to patients and does not require insurance. Tuberculosis screening is a prerequisite for obtaining a visa, and if an individual has active TB, entry into the country will not be granted [7].

#### National policy: the Netherlands

The Netherlands’ policies are dependent on migration type, though treatment is available without charge for all persons notified in the Netherlands. Immigrants from high TB-incidence countries who intend to stay more than 3 months are referred by the immigration office to the municipal health service for screening, which is continued at 6-month intervals for 2 years [1, 12].

Asylum seekers from high TB incidence countries are screened upon arrival at dedicated reception centres.

The Immigration Act stipulates that undocumented migrants with TB can stay in the Netherlands for the duration of their treatment. Housing, health insurance and the costs of living are covered during this period [12].

#### National policy: United Kingdom

In the UK, pre-entry screening for active TB based on clinical evaluation and CXR is required for all individuals applying for a UK visa longer than 6 months. This is accomplished through a pre-emigration procedure in the country of origin or transit of the migrant [12].

Currently, asylum seekers and refugees are entitled to free primary and secondary care. Asylum seekers and undocumented migrants have access to limited NHS care; they are entitled to free primary care at the discretion of the health care provider but have to pay for secondary care. Frequently, they are denied free universal access to TB care [20, 28] .

#### National policy: United States

Unlike the other countries in this review, the United States does not have a universal healthcare system, which limits access to non-citizen migrants, particularly those without employment or legal status [12]. Though the Advisory Council for the Elimination of Tuberculosis (ACET), which does not implement official US regulations, recommends that a lack of health insurance should not be a condition for the timely initiation of appropriate TB treatment it is not clear how this policy is implemented for migrants, particularly the undocumented, across states. The ACET recommendations also state that state health departments can request reimbursement from third-party payers [29]. Following the passage of the Affordable Care Act, which guaranteed TB care to all citizens as part of a national health coverage scheme, some researchers have noted that this coverage does not extend to undocumented migrant groups [30, 31].

The U. S request for pre-entry examinations for those planning a stay of more than six months [23, 32]. Those declared refugees are eligible for Medicaid, state-funded subsidized insurance, for the first eight months after their arrival in most states, and can assess TB care through this program [23, 32].

### TB incidence among non-citizen migrants

The annual incidence rate for TB among non-citizen migrants declined across all the countries included in the study, between 28.8% in the United Kingdom to only 10.7% decline in the United States. Additional details shown in Table 2.

**Table 2:** Incidence of tuberculosis (per 100,000 people) among non-citizen migrants, by country and by notification year

Country	TB among non-citizen migrants		
	2000	2015	% Decrease
Australia	18.0	16.1	10.5
Israel	40.0	11.4	71.5
The Netherlands	61.0	33.8	44.6
United Kingdom	80.0	51.2	36.0
United States	25.8	15.1	41.5
Ref: [2, 4, 8, 10, 19, 22, 25, 33–35]			

### Characteristics of all TB cases among non-Israeli citizens in Israel

Out of the 6321 cases of TB notified in Israel between 2000 and 2014, 1580 were non-citizen migrants (25.6%). 1556 cases were included in the analysis, and incomplete files were not included (98.5%). The sex ratio for the male to female patients was 2:1. Migrants originated from the Horn of Africa (56.2%), Asia (25.5%), Eastern Europe (7.3%), other sub-Saharan African countries (3.9%), and other countries (7.1%). For most migrants, TB was diagnosed within two years from arrival (70.4%).

### Treatment outcomes of non-citizen migrants

In Israel, the percentage of TB patient who completed treatment was 47.5% and the percentage cured was 39.5%, meaning the overall success rate was 87% (n=1354).

The treatment success rate was 87.9% among non-HIV infected TB patients in contrast to 71.8% among HIV co-infected TB patients (OR: 3.025; CI: 1.582–5.786) (Table 3).

**Table 3:** Overall outcome of treatment according to demographic origin and risk factors of non-Israeli citizens in Israel; 2000–2014

Variable		Treatment Success		Total	OR	95% CI
		No n(%)	Yes n(%)			
Sex	Male	138 (13.2)	905 (86.8)	1043	0.491	[0.315–0.764]
	Female	64 (12.5)	449 (87.5)	513	–	–
	Overall	202 (13.0%)	1354 (87.0%)	1556		
Region	Horn of Africa	63 (7.2)	812 (92.8)	875	4.006	[1.810–8.870]
	Asia	64 (16.1)	333 (83.9)	397	1.096	[0.492–2.439]
	Sub-Saharan	15 (24.6)	46 (75.4)	61	0.880	[0.333–2.325]
	Eastern Europe	40 (35.4)	73 (64.6)	113	0.556	[0.236–1.311]
	Other	20 (18.2)	90 (81.8)	110	–	–
Length of Stay	< 2 yrs	98 (11.7)	743 (88.3)	841	0.829	[0.542–1.269]
	> 2 yrs	42 (11.9)	312 (88.1)	354	–	–
HIV Status	Negative	179 (12.1)	1296 (87.9)	1475	3.025	[1.582–5.786]
	Positive	22 (28.2)	56 (71.8)	78	–	–

Of other countries included in the study, only two could provide data on treatment success for non-citizen migrants; in the Netherlands, 90.7% of TB outcomes were successful (personal communication, Dr. Gerard de Vries, KNCV Tuberculosis Foundation). In the UK, 82.1% of TB outcomes among non-citizen migrants were successful (personal communication, Dr., Maeve Lalor, Public Health England).

## Discussion

A major finding in the literature was the insufficient TB data on non-citizen migrants and the difficulty of stating clearly the distinction between a non-citizen migrant and a foreign-born migrant, and different application of these definitions across countries. Most of the literature focused on data on the foreign-born ignoring the possibility that some foreign-born could be eligible for citizenship on arrival or after several years in the low incidence TB country. This could be partly explained by the logistic difficulties of distinguishing between documented and undocumented migrants, which are often coupled with legal national restrictions concerning identified notifications [24].

In Israel, most non-citizen migrant TB patients originate from the Horn of Africa. This was also similar to the findings of previous studies which detected a significant increase of TB in immigrants who migrate to high-income countries [36]. We observed differences (*p* < 0.05) in the HIV infection and TB in migrants in Israel with migrants from African regions being the highest. Similar findings were seen in a previous study in the UK [36].

From 2000 to 2015, incidence rates of TB among non-citizen migrants fell between 10.5–71.5% in the countries of study. Across all the countries under study, Israel reported the lowest TB incidence rate among migrants and the UK reported the highest at the end of 2015. This low incidence rate was achieved despite Israel having the third lowest incidence rate among migrants in 2000. It’s important to note that, in addition to meeting the pre-elimination target during the years of study in the total population, Israel neared this target in the non-citizen migrant sub-population. Direct comparisons between countries must be made with caution, as the characteristics and health determinants of persons screened vary by both by country of origin and destination.

The successful outcome of TB treatment among non-citizens migrants may be attributable to the policy in various countries. However, not all countries under the current study could provide data on non-citizen migrant treatment outcomes. Countries who could provide this data also had migrant-friendly TB policies, with the most success in the Netherlands, where social assistance is provided to TB patients during their treatment. Additional social welfare may explain why TB treatment outcome in the Netherlands is higher than other countries under study.

WHO has emphasized that TB diagnosis and treatment must be free of charge for all migrants [2, 17]. This has guided some low-incidence countries to have low-cost or free TB treatment. However, problems for migrants to access treatment persist. Some low-incidence countries do not provide free TB care at a national level, as is the case in the US [2, 11]. In the UK, undocumented migrants may be excluded from national health services or insurance schemes, which limits overall service access. Moreover, while access to TB is universally accessible on paper in the UK, other important barriers can restrict access and adherence, including language, stigma, and discrimination [2]. In The Netherlands, where health insurance is available for non-citizen migrants, co-payments can constitute financial barriers for some.

Migrants in the US do not have completely free access to TB care and are provided treatment at a cost to the individual either as co-payment where health insurance is made available or out of pocket spending [12, 33, 37, 38]. This may discourage migrants who do not have a source of income to start treatment when diagnosed with active TB, especially migrants who are not legal residents in the country [2, 33].

In addition to policy and other barriers in the countries in the present study, the WHO notes further barriers for migrants in accessing TB care, including fear of being reported to migration officials and/or deported during treatment [24].

Interestingly, Israel created this policy over a decade before the WHO recommendation. This may greatly contribute to the success of TB control in this country [4, 22, 27]. The percentage of successful treatment outcome in Israel was high, particularly for a population considered difficult to reach [4]. The early implementation of a care-for-all strategy for TB may have contributed to the high rate of successful treatment among non-citizen migrants.

### Limitations of the study

Data quality is primarily the responsibility of every country. The study data from Israel includes secondary data analysis from the National TB program; minimal errors could have occurred during data entry and computations but would not have affected the study’s results. Other countries’ data were from the literature, KNCV, and unpublished data from Public Health England, which were analysed by local experts. Also, the periods of years reviewed were not uniform. Hence, our results might not provide a complete or uniform picture of TB among non-citizen migrants across. Furthermore, TB outcome of treatment among immigrants could not be calculated in some selected countries due to the unavailability of non-citizen migrant population data.

### Strengths of the study

To the best of our knowledge, the study is the first formal effort to articulate TB policy and subsequent treatment outcomes of non-citizen migrants in the low incidence countries.

## Conclusion and recommendations

Lack of data on the outcome of TB treatment among non-citizens in the selected countries is a drawback to describing the relationship of each countries policy with respect to the treatment outcome. The US was the only country under this study that does not have a national policy for universal TB care, though many individual states within the US do extend no or low cost TB care. This study supports that TB policies that extend care to non-citizen migrants, including the undocumented, is essential for TB elimination. Moreover, this study highlighted the difficulties of researching TB among non-citizen migrants, as countries operate on their own definitions of migration and citizen type. There is a global need to standardize the definitions of migrants for research purposes. Israel’s trailblazing policy to provide TB care regardless of citizenship status extended care to a large percentage of the TB case load. Following the Netherlands’ model, TB treatment success among this population in Israel may benefit from additional social assistance during treatment to increase compliance and ability to complete treatment, including housing and cost of living.

## Data Availability

This material could be obtained from the corresponding author if the request is in accordance to the Israeli Ministry of Health requirements.
